# Don’t Take down the Stove

**Published:** 1875-05

**Authors:** 


DON'T TAKE DOWN THE STOVE.—Now that spring is coming on, it is the fashion to take down stoves from parlors and sitting rooms, and thus save the trouble and expense of keeping fires. It is not expected that the ladies will wait until the weather is so warm that their families will be perfectly comfortable without fire. If the season of house-cleaning has arrived, the domestic revolutions therein involved cannot be delayed on account of the weather. Having passed through the tribulation of the times, the house comes forth clean, and sweet, and *cold.* Then there are days and evenings of sitting in chilly rooms; there are the half-shivering sensations that perhaps pass unheeded, but are sure warnings of approaching cold, or cough, or pneumonia. It is much safer to be exposed to "the peltings of the merciless storm," to go through rain and snow out of doors all the day, than to sit for an hour in a room which is considered not cold enough to require a fire, but which is really not warm enough to be exactly comfortable. In this climate it is never safe to dispense with our stoves before the first of June.

---

CHOLERA yields to early treatment, the method of meeting the disease by getting in advance of the premonitory diarrhea having proved very successful. The prophylactic to be employed is simply "sulphuric acid lemonade," administered in daily doses containing half a dram of the dilute acid. It is stated that on one occasion forty inmates of an asylum were suddenly attacked by cholera, seventeen of whom succumbed almost immediately. Sulphuric acid lemonade was ordered for all the inmates (400), and no one else was attacked, the remaining cases making good recoveries. Water for drinking should be "cooked" and flavored with a pinch of tea or coffee, cinnamon, quassia, etc.

---

PINS FOR BABIES.—A very worthy writer of a domestic medical book, enumerates among the articles of a baby's wardrobe, "two papers of pins, large and small." We desire to protest against the barbarous custom of using the common pin in a child's clothing. There are plenty of safety-pins of various devices which are all that their names profess, and which will insure the little one against the injuries so often caused by the old fashioned pin. Tapes and buttons should be used for the more permanent fastenings in the child's clothes. It is cruel to subject a baby to the risk and even certainty of being pricked and scratched by torturing pins, when, by a little care and trouble on the part of the mother or nurse, all this may be avoided.

---

IF a case of diptheria occurs, let the patient be at once taken to the top of the house and into the sunniest room, and there kept in perfect isolation from all other inmates of the house save those who are in attendance, as nurses. Ventilation, fumigation, disinfection, and cleansing could there be concentrated and practiced in the best ways possible under the circumstances for the advantage of the sick. The treatment, with ice and milk, described in a late issue, will, under these circumstancs, save nine cases in every ten, if not a greater proportion.

